# Carbolic Acid as a Local Anæsthetic

**Published:** 1875-07

**Authors:** Eugene Foster

**Affiliations:** Augusta, Ga.


### CARBOLIC ACID AS A LOCAL ANÆSTHETIC.

Augusta, Ga., May 23, 1875.

*Editors Atlanta Medical and Surgical Journal:*

Gentlemen—In looking over the proceedings of the recent meeting of the Georgia Medical Association, published in your Journal, I find the following note of an essay read by me:

"Dr. Eugene Foster read an essay upon carbolic acid as a local anæsthetic in surgical operations. He admits that it is a local anæsthetic, but has found it to be escharotic also, as the skin dies and is thrown off; and it is curious to notice that is followed by no suppuration during the process of cicatrization. It produces great pain in whitlow. Regards it, as a local anæsthetic, to be utterly worthless."

As your reporter was mistaken in reference to the grounds taken by me on this subject, I beg to correct him.

In the essay referred to I admitted that we could produce local anæsthesia with carbolic acid, but contended that escharotic action almost invariably followed its use when complete anæsthesia was obtained. I also contended that if the acid penetrated the deeper tissues in sufficient quantity to produce anæsthesia in them that they too would be destroyed, and that it was quite noticeable that the tissues destroyed did not suppurate except at the line of demarkation.

I did not assert that "it (the acid) produces great pain in whitlow," but used the following language in reference to its use in whitlow: I have used the acid in three instances preparatory to incising whitlow. Every one of these patients complained of the great pain caused by the incision. One of them—a lady—fainted. She informed me that she had previously had one opened, when no anæsthetic was used, and that she was positive that the pain was as great in the present as in the former instance. I regard the anæsthetic action af the acid as wholly worthless in these cases.

I also used the following language: From my experience in its use, I assert that carbolic acid is almost worthless as a local anæsthetic; that if complete anæsthesia of a part is obtained, death of the tissues to the extent of the depth of the anæsthesia almost invariably follows; that it is by no means certain to produce anæsthesia, but that it is merely an experiment in each instance in which it is used; that cicatrization of the incision is greatly retarded by the acid; and that in comparison with the ice and salt mixture, ether or chloroform as local anæsthetics, it sinks into utter insignificance. I also had the boldness to prophesy that carbolic acid never could or would be accepted by the profession as a safe and reliable local anæsthetic agent.

As my paper read at the meeting of the Georgia Medical Association is the only one known to me which denies to carbolic acid the right to a place in the class of local anæsthetics, you will greatly oblige me by giving this letter to the public through your JOURNAL. Very respectfully,

EUGENE FOSTER, M.D.

